# Urocortin 3 signalling in the auditory brainstem aids recovery of hearing after reversible noise‐induced threshold shift

**DOI:** 10.1113/JP278132

**Published:** 2019-07-24

**Authors:** Matthew J. Fischl, Margarete A. Ueberfuhr, Markus Drexl, Sara Pagella, James L. Sinclair, Olga Alexandrova, Jan M. Deussing, Conny Kopp‐Scheinpflug

**Affiliations:** ^1^ Department of Biology II Division Neurobiology Ludwig‐Maximilians‐University Munich Germany; ^2^ German Center for Vertigo and Balance Disorders University Hospital Munich Ludwig‐Maximilians‐University Munich Germany; ^3^ Graduate School of Systemic Neurosciences Ludwig‐Maximilians‐University Munich Germany; ^4^ Max Planck Institute of Psychiatry Molecular Neurogenetics Munich Germany

**Keywords:** acoustic trauma, neuropeptide, urocortin, Corticotropin‐releasing hormone receptor, auditory brainstem responses, ageing, DPOAEs, stress recovery

## Abstract

**Key points:**

- Ongoing, moderate noise exposure does not instantly damage the auditory system but may cause lasting deficits, such as elevated thresholds and accelerated ageing of the auditory system.
- The neuromodulatory peptide urocortin‐3 (UCN3) is involved in the body's recovery from a stress response, and is also expressed in the cochlea and the auditory brainstem.
- Lack of UCN3 facilitates age‐induced hearing loss and causes permanently elevated auditory thresholds following a single 2 h noise exposure at moderate intensities.
- Outer hair cell function in mice lacking UCN3 is unaffected, so that the observed auditory deficits are most likely due to inner hair cell function or central mechanisms.
- Highly specific, rather than ubiquitous, expression of UCN3 in the brain renders it a promising candidate for designing drugs to ameliorate stress‐related auditory deficits, including recovery from acoustic trauma.

**Abstract:**

Environmental acoustic noise is omnipresent in our modern society, with sound levels that are considered non‐damaging still causing long‐lasting or permanent changes in the auditory system. The small neuromodulatory peptide urocortin‐3 (UCN3) is the endogenous ligand for corticotropin‐releasing factor receptor type 2 and together they are known to play an important role in stress recovery. UCN3 expression has been observed in the auditory brainstem, but its role remains unclear. Here we describe the detailed distribution of UCN3 expression in the murine auditory brainstem and provide evidence that UCN3 is expressed in the synaptic region of inner hair cells in the cochlea. We also show that mice with deficient UCN3 signalling experience premature ageing of the auditory system starting at an age of 4.7 months with significantly elevated thresholds of auditory brainstem responses (ABRs) compared to age‐matched wild‐type mice. Following a single, 2 h exposure to moderate (84 or 94 dB SPL) noise, UCN3‐deficient mice exhibited significantly larger shifts in ABR thresholds combined with maladaptive recovery. In wild‐type mice, the same noise exposure did not cause lasting changes to auditory thresholds. The presence of UCN3‐expressing neurons throughout the auditory brainstem and the predisposition to hearing loss caused by preventing its normal expression suggests UCN3 as an important neuromodulatory peptide in the auditory system's response to loud sounds.

## Key points

- Ongoing, moderate noise exposure does not instantly damage the auditory system but may cause lasting deficits, such as elevated thresholds and accelerated ageing of the auditory system.
- The neuromodulatory peptide urocortin‐3 (UCN3) is involved in the body's recovery from a stress response, and is also expressed in the cochlea and the auditory brainstem.
- Lack of UCN3 facilitates age‐induced hearing loss and causes permanently elevated auditory thresholds following a single 2 h noise exposure at moderate intensities.
- Outer hair cell function in mice lacking UCN3 is unaffected, so that the observed auditory deficits are most likely due to inner hair cell function or central mechanisms.
- Highly specific, rather than ubiquitous, expression of UCN3 in the brain renders it a promising candidate for designing drugs to ameliorate stress‐related auditory deficits, including recovery from acoustic trauma.

## Introduction

Protection from acoustic trauma is an important feature of the auditory system that helps to preserve our hearing over decades of life. Recent studies have shown that even moderate noise levels that were previously considered to be non‐damaging can effectively speed up the ageing of the auditory system and cause permanent changes in the physiology of auditory circuits (Gourevitch *et al*. 2014; Hickox & Liberman, 2014).

So far, two main types of protective feedback pathways have been described in the auditory system: the olivocochlear system and the middle ear reflex. The olivocochlear system has two parts: the medial olivocochlear system (MOC) controls the level of the outer hair cells’ contribution to cochlear amplification, and the lateral olivocochlear system (LOC) modulates the excitability of the auditory nerve (Guinan, 2011; Elgoyhen & Katz, 2012; Liberman *et al*. 2014). MOC feedback is mediated by myelinated fibres that can be recorded from, as well as electrically stimulated (for review see Guinan, 2011; Elgoyhen & Katz, 2012). The non‐myelinated LOC fibres are much harder to study and thus to date the physiology and the components involved of this feedback mechanism are less well understood. Both, the MOC and the LOC system use acetylcholine as their main transmitter and so immunostaining for acetylcholinesterase is typically employed to label these efferent neurons (Darrow *et al*. 2006). For some frequency ranges, the middle ear reflex circuit is another feedback loop which acts to reduce the sound energy reaching the cochlea by stiffening the muscles holding the ossicles, ultimately reducing the movement of the stapes in response to incident sound (reviewed in Mukerji *et al*. 2010). An additional, yet uncharacterized feedback mechanism has been postulated and is based on the corticotropin‐releasing factor receptors (CRF‐Rs) and a group of their ligands called urocortins (UCNs) (Vetter *et al*. 2002; Graham *et al*. 2011; Maison *et al*. 2012). It has been shown that increasing corticosteroids systemically following restraint stress protects against noise‐induced hearing loss (Wang & Liberman, 2002). Corticotropin releasing factor (CRF) specifically binds to the CRF‐R1s, which are abundantly expressed in the cochlea where they are suggested to act as a local hypothalamic–pituitary–adrenal (HPA) axis equivalent signalling system (Graham & Vetter, 2011). An efferent projection from LOC neurons of the lateral superior olive to the inner hair cells (IHCs) provides a mixture of neuromodulatory transmitters including acetylcholine, and UCN1 which also binds to CRF‐R1s (Kaiser *et al*. 2011). Compared to the CRF‐R1 receptor, the CRF‐R2 receptor is highly selective for urocortin‐3 (UCN3) (Hsu & Hsueh, 2001; Lewis *et al*. 2001) and is strongly expressed throughout the cochlea particularly in close proximity to the IHC afferent synapse (Graham *et al*. 2010). Graham *et al*. (2011) demonstrated that naïve CRF‐R2 knockout mice were more sensitive to sound, but paid for this gain in function with a higher susceptibility to acoustic trauma. However, to date it is unknown if the neuromodulatory peptide UCN3 is present in the cochlea. UCN3, also known as stresscopin, is the most recently discovered member of the corticotropin‐releasing factor family and mediates the adaptation and recovery mode of the body's stress system (de Kloet *et al*. 2005). UCN3 is expressed in a small number of distinct brain nuclei: the median preoptic nucleus, the rostral perifornical region of the hypothalamus, the medial amygdala, the bed nucleus of the stria terminalis and the superior paraolivary nucleus (SPN) (Lewis *et al*. 2001; Li *et al*. 2002; Wittmann *et al*. 2009; Deussing *et al*. 2010). Most of these central sites of UCN3 expression are associated with modulating stress‐related autonomic, neuroendocrine and behavioural function (Lewis *et al*. 2001; Deussing *et al*. 2010), leaving open the question of the functional significance of UCN3 expression in auditory brainstem neurons for homeostasis and normal ageing in the auditory system.

In the present study, we investigate whether UCN3 expression outside the hypothalamic–pituitary–adrenal axis helps to protect the cochlea during exposure to moderate noise. We use *in vivo* recordings of auditory brainstem responses (ABRs) and measurements of distortion products of otoacoustic emissions (DPOAEs), before and following acoustic exposure to moderate noise in wild‐type mice and in mice lacking UCN3 signalling.

## Methods

### Ethical approval

All experimental procedures were carried out in compliance with German animal welfare law (Tierschutzgesetz), approved by the regional authority (Regierung von Oberbayern) under file no. 55.2‐1‐54‐2532‐38‐13) and conform to the principles and regulations as described in the editorial by Grundy (2015). Experiments were conducted on UCN3 knockout mice (UCN3 KO), UCN3 reporter mice (UCN3 tdTom) and C57BL/6 wild‐type (WT) mice of both sexes. UCN3 KO mice were described previously (Deussing *et al*. 2010). Reporter mice were generated by breeding UCN3‐Cre mice (Tg(Ucn3‐cre)KF31Gsat; The Gene Expression Nervous System Atlas (GENSAT) Project; Deussing *et al*. 2010) with R26^CAG::loxP‐STOP‐loxP‐tdTomato^ mice (Ai9, The Jackson Laboratory, Bar Harbor, ME, USA; stock no: 007905) as previously described (Shemesh *et al*. 2016). UCN3 KO mice and UCN3‐tdTomato mice were on a C57BL/6 background for more than 20 generations and were therefore compared to wild‐type C57BL/6 mice. In addition, to rule out any background strain effects, littermate comparisons were made in the UCN3 knockdown experiments. Mice were housed in a vivarium with a normal light–dark cycle (12 h light/12 h dark) and food and water *ad libitum*.

### Immunohistochemistry

Mice received an overdose of pentobarbital (400 mg kg^−1^ body weight; i.p.) and were perfusion‐fixed with 4% paraformaldehyde (PFA) intracardially. Following overnight postfixation in 4% PFA, brainstems were sectioned coronally at 50 µm using a vibrating blade microtome (V1200S, Leica, Wetzlar, Germany). After rinsing in phosphate‐buffered saline (PBS), sections were transferred to a blocking solution containing 1% bovine serum albumin, 0.5% Triton X‐100 and 0.1% saponin in PBS. The floating sections were then incubated for 48 h at 4°C in blocking solution containing primary antibodies against calbindin (Merckmillipore, Darmstadt, Germany; 1:500). Sections were washed three times in PBS for 15 min and incubated with secondary antibodies (Thermo Fisher Scientific, Munich, Germany; A‐11034 anti‐rabbit Alexa 488, 1:200) overnight at 4°C. Sections were washed in PBS, mounted on slides and coverslipped with Vectashield mounting medium (Vector Laboratories, Burlingame, CA, USA).

### Cochlear whole mount preparation

After perfusion‐fixation (see above), temporal bones were removed and post‐fixed in 4% PFA overnight. Following post‐fixation, the temporal bones were decalcified in 4% EDTA (pH adjusted to 7.4 with NaOH) for several days. Cochlear tissue including the organ of Corti was isolated and processed using immunohistochemical methods (see above). After immunohistochemical staining, tissue was treated with Phalloidin‐Atto 488 (Sigma‐Aldrich, Taufkirchen, Germany; 49409‐10NMOL Lot BCBN7314V, 1:50) and 4′,6‐diamidino‐2‐phenylindole (DAPI; Thermo Fisher Scientific 62248 lot QI20604220, 1:10 000) for 20 min, followed by two 10 min washes in PBS before coverslipping.

### Cochlear cryostat sectioning

After perfusion‐fixation (see above) and decalcification, cochleae were bisected through the modiolus and embedded in Tissue Tek. Cryostat sections were cut at 12 µm and mounted on Superfrost Plus (Thermo Fisher Scientific) slides. Immunocytochemistry using antibodies for phalloidin and DAPI was performed directly on the slide at the concentrations described above.

### Auditory brainstem response recordings

Auditory brainstem responses were recorded in mice anaesthetized with an i.p. injection of MMF (medetomidin 0.5 mg kg^−1^, midazolam 5 mg kg^−1^, fentanyl 0.05 mg kg^−1^). During the experiments, mice were placed on a temperature‐controlled heating pad (ATC1000, WPI, Sarasota, FL, USA) in a soundproof chamber (Industrial Acoustics, Niederkrüchten, Germany). Disposable needle electrodes (Rochester Electro‐Medical, Inc., Coral Springs, FL, USA) were placed subdermally at the vertex of the mouse's head (reference), ventral to the pinna (active (−)) and near the base of the tail (ground). Electrodes were attached to a low impedance headstage (RA4LI, Tucker Davis Technologies, Alachua, FL, USA) and connected to the auditory processor (RZ6, Tucker Davis Technologies) with an optical input cable through a preamplifier (RA16PA, Tucker Davis Technologies; amplification factor: 250). SPIKE software (Brandon Warren, University of Washington) was used to calibrate the speakers in situ (MF1, Tucker Davis Technologies), generate stimuli and record ABR waveforms. Stimuli consisted of broadband clicks (100 µs duration) or pure tones (4–44 kHz, 5 ms duration) presented 1000× at varying intensities (0–85 dB SPL, 2.5 dB steps). For individual frequencies, responses to the 1000 stimulus repetitions were averaged and thresholds were determined as the sound intensity at which at least two peaks could be distinguished in the average ABR waveform. If no threshold was detected at 85 dB SPL, a value of 90 dB SPL was given as threshold. When possible, the genotype of the animal was blinded to the experimenter (viral knockdown experiments); otherwise, the thresholds were assessed offline by a second person who then was blinded to the genotype. At the end of the experiment, animals were either revived using an i.p. injection of the MMF antidote AFN (atipamezol 2.5 mg kg^−1^, flumazenil 0.5 mg kg^−1^, naloxone 1.2 mg kg^−1^) or they received an overdose of pentobarbital (400 mg kg^−1^ body weight; i.p.) following the last measurement at the 14 day post‐exposure time point.

### Ageing study

Auditory thresholds were assessed by ABR recordings in wild‐type (WT) and UCN3 KO mice aged between 1 month and 2 years. For the ageing study, each animal received an overdose of pentobarbital (400 mg kg^−1^ body weight; i.p.) immediately after ABR measurement; therefore, repeated measures were not obtained for this part of the study. The average age was not significantly different between genotypes (WT: 7.64 ± 0.55 SEM months, *n* = 90; UCN3 KO: 6.58 ± 0.53 months, *n* = 69; two‐tailed *t* test: *P* = 0.175). ABR thresholds of WT and UCN3 KO mice were plotted against the animals’ age and the data were fitted by an exponential rise to maximum function: *f* = *a*(1 − exp(−*bx*)). These fits suggested differences between the two datasets occurring with increasing age. A change detection algorithm (Taylor, 2000) was employed to test whether the difference between WT and UCN3 KO ABR thresholds over time was randomly occurring or statistically significant. It also served to determine the age at which the two datasets started to differ. For this, the cumulative sum of the difference data points (interpolated UCN3 KO ABR thresholds – interpolated WT ABR thresholds) from which the mean of the full time series was subtracted was calculated. Then, the difference between the maximum and the minimum of the cumulative sum time series was determined. A bootstrap analysis (10,000 samples) was used to randomly re‐order the time series and the analysis described before was repeated for each of the re‐ordered samples. The confidence level was then determined by calculating the percentage of 10,000 bootstrap samples where the difference between the maximum and minimum of the bootstrapped cumulative time series was smaller than in the original time series. We considered changes in the original time series significant when the confidence level was equal to or larger than 99%.

### Noise exposure

Bandpass filtered noise between 8 and 16 kHz was generated using RPvdsEx (Tucker Davis Technologies) and was presented bilaterally via earphones (MF1 Magnetic Speakers, Tucker‐Davis Technologies) for 2 h at a level of 94 or 84 dB SPL. During the noise exposure, animals were under MMF anaesthesia (see above) and placed on a temperature‐controlled heating pad (ATC1000, WPI) in a soundproof chamber (Industrial Acoustics). ABRs were recorded before noise exposure (naïve), immediately after (= day 0), 1 day later, 7 days later, and 14 days later (see above, ‘Auditory brainstem response recordings’). Recovery of auditory thresholds was tested against a baseline of threshold variability acquired from repeated ABR measures of six animals at multiple time points (similar to those of the noise‐exposed mice) without acoustic exposure. These data gave us a baseline for threshold change significance testing and showed that our ABR protocol did not induce threshold shifts over the same time course as our noise exposure experiments. The average change in threshold for these control animals without noise exposure was −0.4 ± 1.0 dB SPL. This baseline variability is represented as horizontal grey shaded areas in Figs 3 *F* and *I* and 5 *D*. Threshold shifts in dB were calculated by taking the threshold at each time point and subtracting the threshold in the naïve condition. Animals of each genotype did not significantly differ in age at the time of noise exposure (median (interquartile range): WT: 3.64 (3.48–3.97) months, *n* = 15; UCN3 KO: 4.33 (3.41–4.56) months, *n* = 15; Mann–Whitney rank sum test: *P* = 0.575).

### Distortion product otoacoustic emission recordings

DPOAEs were recorded in C57BL/6J wild‐type mice and UCN3 KO mice. Mice (20–24 g BW, aged 3–4 months) were anaesthetized with MMF (see above) and physiological temperature was maintained by a heating pad during the experiments. All DPOAEs were recorded in a soundproof chamber. Sound signals to elicit DPOAEs were generated and recorded with an external sound card, RME Fireface UC 24‐bit (RME, Audio AG, Haimhausen, Germany) at a sampling rate of 192 kHz and controlled by custom‐written MATLAB scripts (The MathWorks, Natick, MA, USA). To use low‐latency multi‐channel ASIO, the SoundMexPro application (HörTech, Oldenburg, Germany) was employed for interfacing in the MATLAB environment. A custom‐made acoustic coupler (based on the Eaton‐Peabody‐Laboratory (EPL) Acoustic System) consisting of a microphone (FG‐23329‐P07, Knowles, Itasca, IL, USA) and two micro dynamic speakers (CDMG15008‐03A, CUI, Tualatin, OR, USA) was inserted into the ear canal of anaesthetized animals under visual control such that the tip of the coupler was at a distance of about 1 mm from the tympanic membrane.

Before conducting the experiments, the amplitude and phase response of the probe microphone was corrected by comparing it to the flat amplitude and phase response of a reference microphone (B&K, Type 4135, Brüel & Kjær Sound & Vibration Measurement A/S, Nærum, Denmark). Before each trial, the two loudspeakers generating DPOAE primary tones were calibrated *in situ* to guarantee a flat frequency and phase response at the tip of the acoustic coupler. For that, the ear canal was stimulated with band‐pass filtered white noise (0.2–40 kHz) and a compensational impulse response was calculated for each loudspeaker. This compensational impulse response was used to correct all acoustic stimuli delivered by the loudspeakers.

To evoke DPOAEs, two primary tones *f*_1_ and *f*_2_ (*f*_2_/*f*_1_ = 1.23), with *f*_1_ level set to 65 dB SPL and *f*_2_ level set to 55 dB SPL, were presented 32 times with a stimulus duration of 500 ms. The frequency ratio of the primaries (*f*_2_/*f*_1_) and the intensity level were held constant while eliciting DPOAEs at several different frequencies with *f*_2_ between 4 and 40 kHz increasing in increments of 4 kHz steps. Thereby, so called DP‐grams, DPOAE magnitude as a function of and *f*_2_, were generated. The cubic DP (2*f*_1_ −  *f*_2_) was extracted from the ear canal sound pressure after time‐domain averaging. By averaging the magnitudes of six spectral lines below and six above the respective cubic DP, the noise floor was calculated. DPOAE recordings with DPOAE levels less than 6 dB above noise floor were not included in the analysis and rejected. At the end of the experiment, animals were either revived using an i.p. injection of the antidote AFN (atipamezol 2.5 mg kg^−1^, flumazenil 0.5 mg kg^−1^, naloxone 1.2 mg kg^−1^) or they received an overdose of pentobarbital (400 mg kg^−1^ body weight; i.p.) following the last measurement at the 14 day post‐exposure time point.

### Viral vector‐mediated knockdown

UCN3‐tdTomato mice were injected with rAAV8/EF1α‐mCherry‐Flex‐dtA (AAV‐DTA) on postnatal day 22–23 (*n* = 26 animals/3 litters) under MMF anaesthesia (see above) using a Neurostar stereotaxic injection system (Tübingen, Germany). The superior olivary complex (SOC) was targeted at the interaural midline level (about 5–6 mm caudal of Bregma) and 0.5 µl of the viral vector solution was injected slowly over the time course of 3 min. This virus is taken up by all neurons, but only those neurons expressing the enzyme cre‐recombinase (in our experiment only those that transcribe UCN3) will produce diphtheria toxin and subsequently degenerate. At the end of the injection, mice were injected with the analgesic Metacam (1–5 mg kg^−1^, s.c.). The animals were then sutured and the AFN antidote (see above) was given to terminate anaesthesia. After the operation, each animal was placed in a single cage, which was irradiated by an infrared heat lamp, and continuously observed until the animal has recovered to the point where it has full motor coordination and begins normal feeding and grooming behaviour. At that point the animal was united with its original group. So far, we have had good experiences with this form of return to the group: even in males, there were no complications with re‐integration. During post‐operative recovery the animals received an injection of Metacam every 24 h for 3 days. The health status of the animals was evaluated daily for at least the next 5 days using a score sheet including body weight, posture, reaction to handling, physical appearance as well as motor and social behaviour. All animals used in this study had a total score of zero on day 5, rendering them comparable to untreated mice in the colony; 28 to 49 days were allowed for expression of diphtheria toxin, before the ABR and noise exposure protocols were then performed as described in previous paragraphs. After the 2 week time point, animals received an overdose of pentobarbital (400 mg kg^−1^ body weight; i.p.), were perfused intracardially, and the brains were examined following sectioning to assess the cre^+^ or cre^−^ genotype. Note, that up until this point the experimental group (cre^+^: showing reduced UCN3 expression in the auditory brainstem) and the control group (cre^−^: WT with normal UCN3 expression) were completely blinded to the experimenter. Animals of each genotype did not differ in age at the time of noise exposure (cre^+^: 57.3 ± 1.5 days, *n* = 13 animals; cre^−^: 60.5 ± 1.7 days, *n* = 13 animals; two‐tailed Student's *t* test: *P* = 0.167). Tissue sections were counterstained with microtubule‐associated protein 2 (MAP2; primary: chicken anti‐MAP2, Acris (Herford, Germany) CH22103, 1:1000; secondary: donkey anti‐chicken aminomethylcoumarin acetate (AMCA), 703‐156‐155 Dianova, Hamburg, Germany, 1:100) following the protocol above.

### Experimental design and statistical analysis

In the text, data are presented as median (interquartile range) or as mean ± SEM. In the figures, data are presented as medians (lines in boxes); 25/75 quartiles (boxes); and 10/90 percentiles (whiskers) in addition to individual data points. Statistical analyses of the data were performed with SigmaStat/SigmaPlot (Systat Software, Inc., San Jose, CA, USA). Normality was tested by the Shapiro–Wilk test. Comparisons between different datasets were made depending on the distribution of the data using parametric tests for normally distributed data (two‐tailed Student's *t* test for comparing two groups and ANOVA for comparing three or more groups). When the normality assumption was violated, non‐parametric tests (Mann–Whitney rank sum test for comparing two groups and Kruskal–Wallis ANOVA on ranks for comparing three or more groups) were used. A paired *t* test or Mann–Whitney rank sum test was used when two data sets were compared in before–after conditions. Differences were considered statistically significant at *P* ≤ 0.05 and presented in the figures as n.s. for non‐significant differences and as ^*^ *P* ≤ 0.05, ^**^ *P* ≤ 0.01, ^***^ *P* ≤ 0.001 for significant differences. In the test all actual *P*‐values are given for values >0.001.

## Results

### UCN3 is expressed in neurons of the auditory brainstem and synaptic terminals in the cochlea

**Figure 1:**
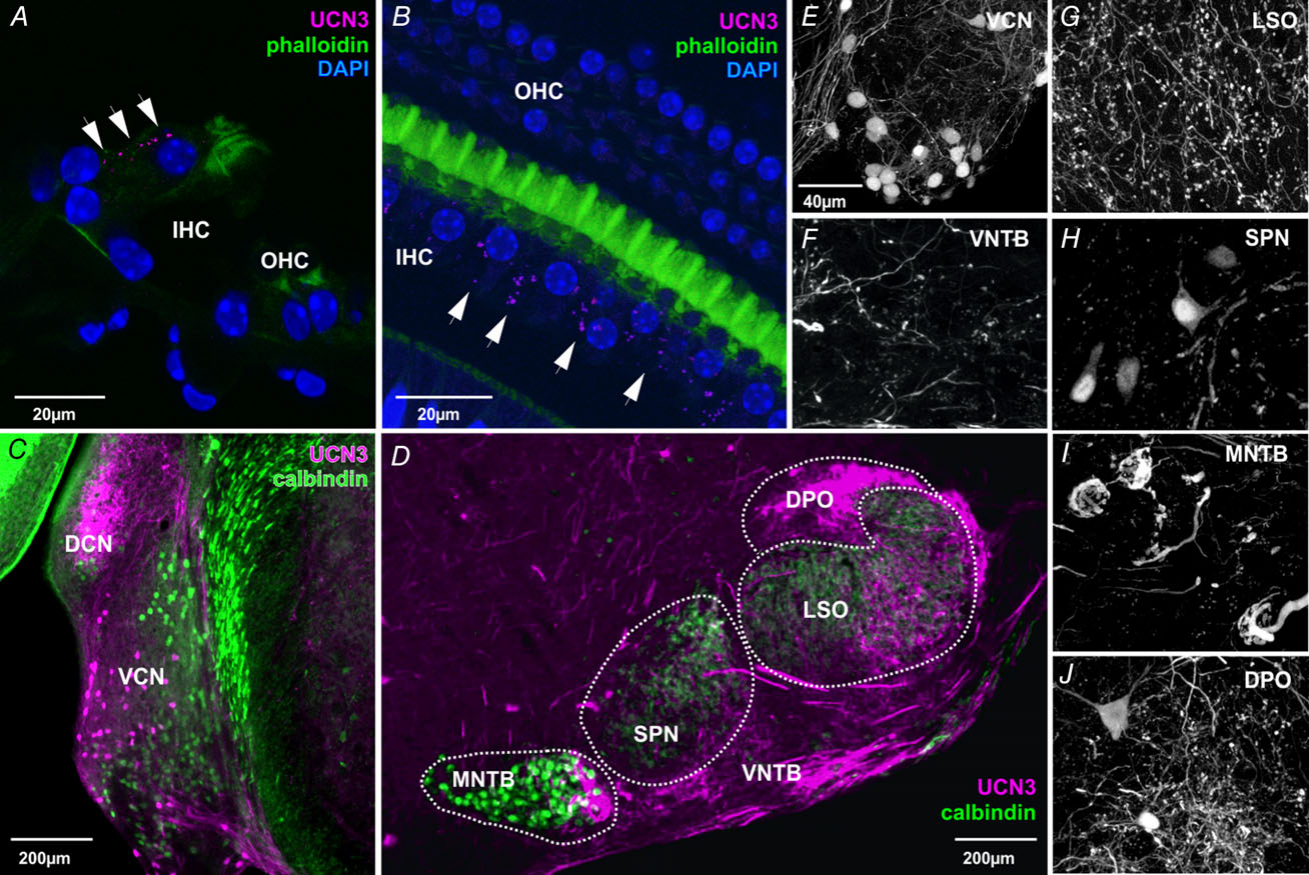
UCN3 reporter mouse line shows expression of UCN3 in the cochlea and the auditory brainstem *A*, cryostat cross section (12 µm) of the organ of Corti of UCN3‐tdTomato mouse, with DAPI (blue), phalloidin (green) and tdTomato (magenta) fluorescence. UCN3 positive puncta were present at the modiolar side of the inner hair cell (IHC) as indicated by tdTomato fluorescence (white arrows). *B*, whole mount preparation of the cochlea of UCN3‐tdTomato mouse. Outer hair cells (OHC) and IHCs are identified by staining their nuclei (DAPI: blue) and the staining of surrounding pillar cells (phalloidin: green). UCN3 positive puncta (magenta) were present at the base of the IHCs (white arrows). *C*, coronal brainstem section containing the dorsal (DCN) and ventral (VCN) cochlear nucleus. tdTomato fluorescence (magenta) labels neuronal somata in the VCN and terminal fields in the DCN. Calbindin (green) served as a counterstain. *D*, coronal brainstem section containing the nuclei of the SOC. *E*–*J*, high magnification images of UCN3‐expressing neurons and terminals depicted in *C* and *D*. DPO, dorsal paraolivary nucleus; LSO, lateral superior olive; MNTB, medial nucleus of the trapezoid body; SPN, superior paraolivary nucleus; VNTB, ventral nucleus of the trapezoid body.

UCN3 is a small neuromodulatory peptide expressed in the brainstem as evidenced by *in situ* hybridization and genetic labelling (Lewis *et al*. 2001; Li *et al*. 2002; Deussing *et al*. 2010). Here, we took advantage of a UCN3‐tdTomato reporter line in order to determine the pattern of UCN3 expression in the cochlea and the auditory brainstem (Fig. 1). Fidelity of the reporter has been previously confirmed (Shemesh *et al*. 2016). Figure 1 *A* shows a cross section of the organ of Corti with tdTomato fluorescent puncta present near the base of the IHCs (white arrows). Additionally, cochlear whole mount sections of UCN3‐tdTomato reporter mice were prepared and counterstained with phalloidin and DAPI to visualize the surrounding pillar cells and nuclei of the auditory hair cells, respectively, alongside UCN3 expression as indicated by the tdTomato fluorescence signal (Fig. 1 *B*). The image shows three rows of outer hair cells (OHCs) and one row of IHCs. Again, UCN3 positive puncta were observed at the base of the IHCs (white arrows), resembling the pattern of the afferent ribbon synapses (Sergeyenko *et al*. 2013).

In the brainstem, we observed UCN3 expression in neuronal somata, axons and dendrites of the ventral cochlear nucleus (VCN) and neuropil staining in the dorsal cochlear nucleus (DCN; Fig. 1 *C* and *E*). From the UCN3‐expressing neurons in the VCN, labelled fibres project towards the DCN, which suggests at least some of these cells are multipolar cells (Doucet & Ryugo, 1997). In addition, UCN3 positive fibres leave the VCN via the ventral acoustic stria and form synaptic terminals in the lateral division of the contralateral medial nucleus of the trapezoid body (MNTB), the ventral nucleus of the trapezoid body (VNTB) and the lateral superior olive (LSO; Fig. 1 *D*, *F*, *G* and *I*). The presence of UCN3 positive calyx of Held synapses in the lateral MNTB (Fig. 1 *I*) implies that a subpopulation of the UCN3‐expressing neurons in the VCN are globular bushy cells, which are known to give rise to calyx of Held synapses (Spirou *et al*. 1990). The calcium‐binding protein calbindin is a helpful marker for auditory brainstem neurons and was used here as a counterstain (Zettel *et al*. 1991). Within the SOC, UCN3‐expressing neuronal somata were observed in the superior paraolivary nucleus (SPN) at the dorso‐lateral border of the MNTB (Fig. 1 *E* and *H*). The dorsal periolivary nucleus (DPO) contains a rich network of UCN3‐expressing fibres and neuronal somata (Fig. 1 *D*, *H* and *J*). Interestingly both of these para‐ (in guinea pig) and periolivary (in several other rodent species) nuclei are also host to neurons of the efferent feedback system to the cochlea (Thompson & Thompson, 1991; Warr *et al*. 1997).

### UCN3 KO mice demonstrate a more rapid progression of age‐related hearing loss than C57BL/6 WT

**Figure 2:**
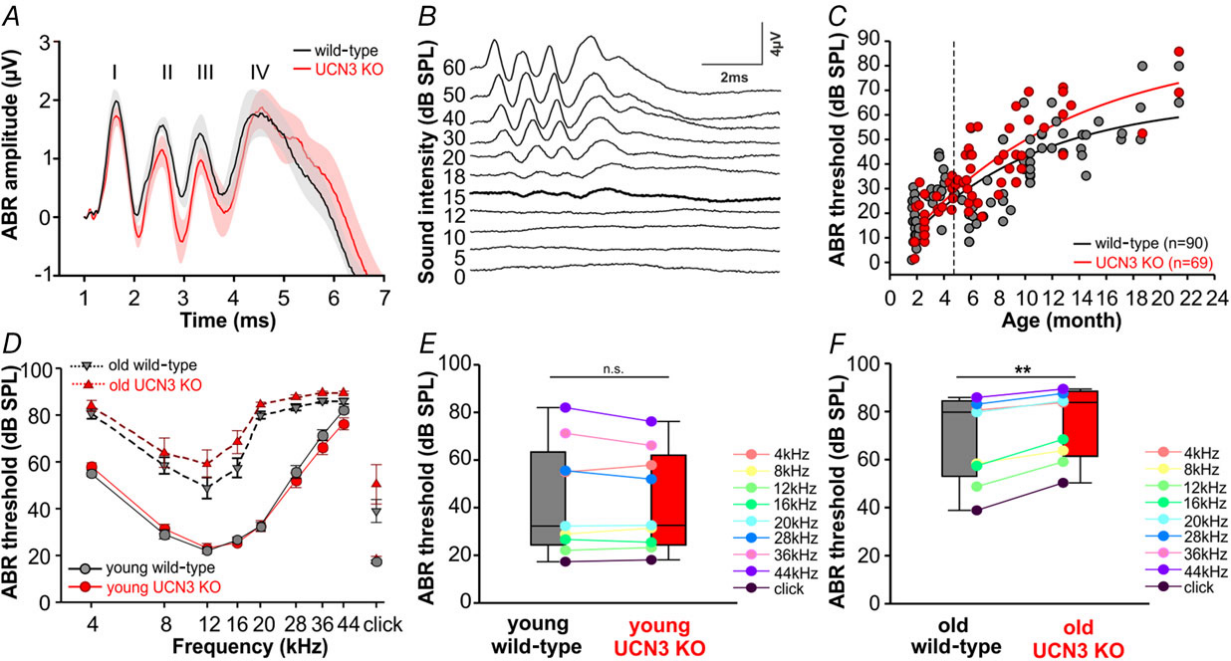
Auditory thresholds of UCN3 KO mice increase faster than wild‐type as a function of age *A*, grand average and SEM of ABR recordings in response to 80 dB SPL click stimuli for WT (black) and UCN3 KO (red) mice showed 4 clearly distinguishable peaks (I–IV). *B*, typical ABR recording in response to click stimuli of decreasing (top to bottom traces) intensities. Threshold is indicated by the thicker line (here 15 dB SPL). Each line is an average generated from responses to 1000 stimulus presentations. *C*, ABR threshold of WT (black) and UCN3 KO (red) mice plotted against age. Continuous lines depict exponential growth to maximum fit for WT (black) and UCN3 KO (red). The vertical dashed line delineates the age at which the thresholds of UCN3 KO mice deviate significantly from those of wild‐types. *D*, audiograms for young and older mice for WT (grey) and UCN3 KO mice (red). Note the threshold elevation for high frequencies (28–44 kHz) even in young mice (continuous lines) of both genotypes, typical for the C57Bl/6 background. *E* and *F*, ABR thresholds averaged across frequencies for the WT and UCN3 KO mice younger (*E*) and older (*F*) than 4.73 months (dashed line in *C*). Individual frequencies are colour‐coded and paired between genotypes.

Different mouse strains show distinct susceptibility to age‐induced hearing loss with the C57BL/6 strain being especially prone (Henry & Chole, 1980). Since the UCN3‐tdTomato reporter mice and UCN3 knockout (UCN3 KO) mice that were used in this study were generated in a C57BL/6 background, it was important to verify up to what age hearing thresholds in these mice were sensitive enough for the subsequent sound exposure experiments. Auditory brainstem response (ABR) recordings were performed over a broad age range (from 1 month to 2 years) in C57BL/6 WT and UCN3 KO animals to determine an age cutoff before significant age‐induced hearing loss would occur. Figure 2 *A* shows the average ABR response to a click stimulus at 80 dB SPL, for both WT (black) and UCN3 KO (red) populations. Presentations of different sound intensities were used to determine auditory thresholds (Fig. 2 *B*). ABR thresholds were plotted against each animal's age in order to estimate if and at what age hearing thresholds of UCN3 KO mice deviate significantly from the WT mice. The progressive elevation of ABR thresholds with increasing age could be predicted by fitting exponential rise to maximum functions to each dataset (Fig. 2 *C*). These estimates suggested a faster elevation of ABR thresholds with increasing age in UCN3 KO mice. The difference in ABR thresholds between WT mice and UCN3 KO mice became significant at 4.73 months (dashed vertical line in Fig. 2 *C*; see Methods for details on analysis), which therefore became the age of division between young and old mice. Audiograms comparing young and old animals of each genotype are depicted in Fig. 2 *D*. Young animals of UCN3 KO and WT mice have similar and overlapping thresholds, but old UCN3 KO animals tended to have higher ABR thresholds than aged WT mice at all frequencies. This genotype‐specific elevation of ABR thresholds was quantified across the audiogram and revealed no statistically significant difference for young mice (WT: 43.45 ± 7.72 dB SPL; UCN3 KO: 42.55 ± 6.97 dB SPL; two‐tailed *t* test: *P* = 0.425; Fig. 2 *E*). Old UCN3 KO mice had significantly higher ABR thresholds (UCN3 KO: 83.75 (61.41–88.44) dB SPL) compared to age‐matched WT mice (79.72 (53.05–84.44) dB SPL; Wilcoxon signed rank test: *P* = 0.004; Fig. 2 *F*). To remove the confounding effect of differences in age‐induced hearing loss from our subsequent experiments, we used only young animals <4.73 months to study the impact of UCN3 on noise‐induced hearing loss.

### Following noise exposure, UCN3 KO mice exhibit larger threshold shifts and slower recovery than C57BL/6 WT mice

**Figure 3:**
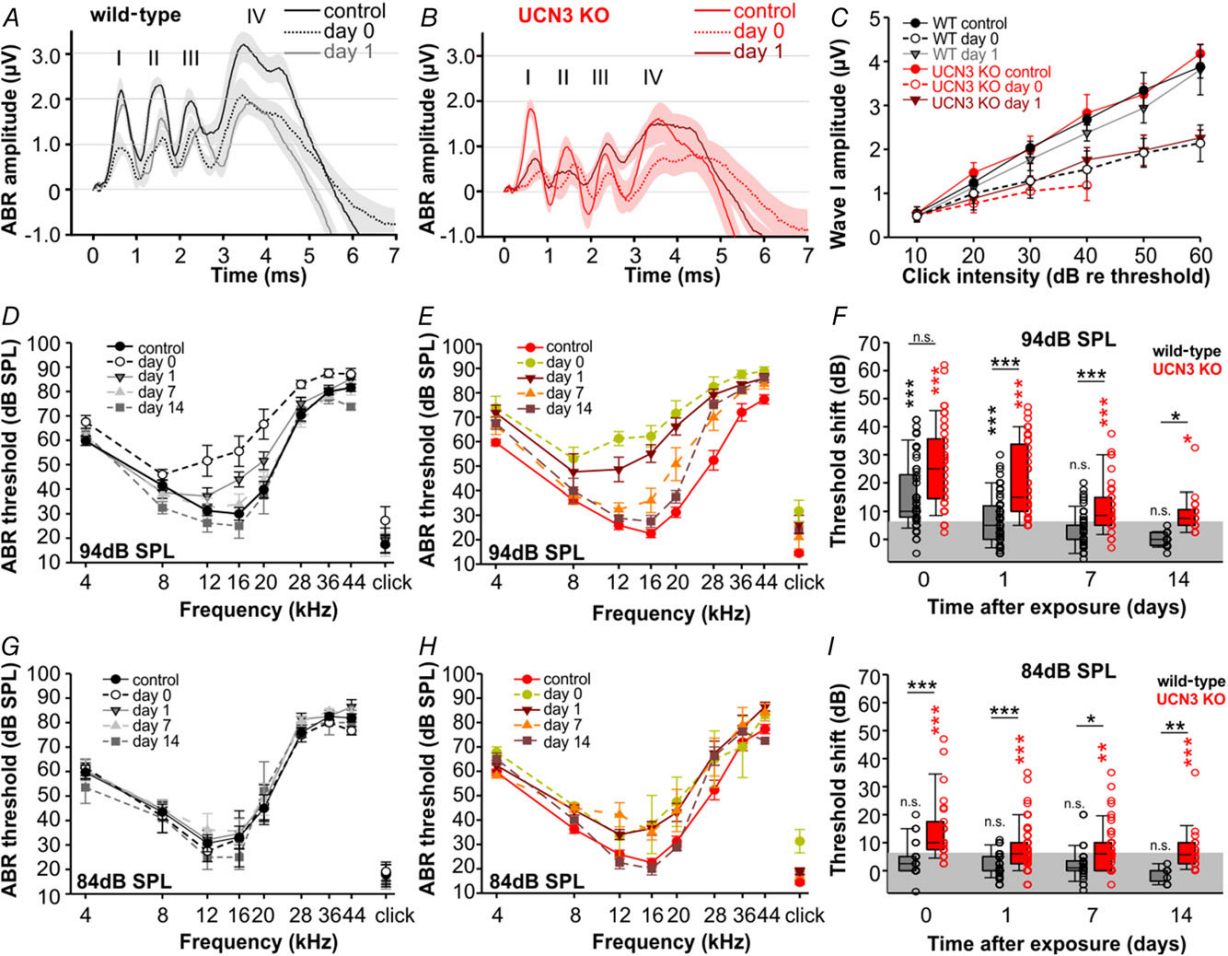
UCN3 KO and WT mice show different threshold shifts and recovery following noise exposure *A* and *B*, grand average and SEM of ABRs before (control), immediately after (day 0) and 24 h (day 1) after a 2 h/94 dB SPL noise exposure for WT (*A*, black, *n* = 15) and UCN3 KO (*B*, red, *n* = 15) mice. *C*, ABR wave I amplitudes for the same genotypes and time points as in *A* and *B* show stronger and longer lasting reduction in UCN3 KOs compared to wild‐type controls. Note, that due to the larger threshold shifts in the UCN3 KO, fewer data points above threshold could be collected especially for day 0. *D* and *E*, shifts in ABR thresholds following a 2 h/94 dB noise exposure in wild‐type (*D*) and UCN3 KO mice (*E*). *F*, threshold shift in dB relative to threshold averaged over all frequencies. Black and red asterisks indicate that a significant difference was detected relative to baseline (shaded area); *P*‐values indicate whether a significant difference was found between genotypes: Kruskal–Wallis one‐way ANOVA on ranks followed by multiple comparisons *versus* Control group (Dunn's method). *G*–*I*, same figure design as *D–F*, but following exposure to 2 h of 84 dB SPL noise.

To investigate the role of the stress peptide UCN3 in the auditory system's response to loud sounds, we adapted a noise exposure protocol that is reported to induce mild acoustic trauma without damaging the peripheral or central auditory system (Hickox & Liberman, 2014). After initial ABR recordings in the naïve condition, animals were exposed to a bandpass filtered noise (8–16 kHz, 94 dB SPL) for 2 h. Following noise exposure, the animals’ ABR thresholds were measured at several subsequent time points: immediately, 1, 7 and 14 days post‐exposure. Figure 3 *A* and *B* shows the average ABR waveforms for each genotype in response to an 80 dB SPL click stimulus before exposure (94 dB SPL noise) and two subsequent time points following exposure. Decreases in wave I amplitude were observed for both genotypes immediately after exposure. However, WT wave I amplitudes recovered to near control amplitudes while UCN3 KO amplitudes remained reduced at the day 1 time point (Fig. 3 *A*–*C*). Waves II–IV seem to show longer lasting effects in both genotypes, which will have to be addressed in a separate study by corresponding cellular physiology in the different processing stages of the ascending auditory pathway. Audiograms for the group of animals exposed to 94 dB SPL noise are shown in Fig. 3 *D* and *E*. Threshold values in the naïve condition were subtracted from threshold values at each post‐exposure time point to obtain a threshold shift value. Threshold shift values were averaged across the audiogram and compared between genotypes as well as against a baseline acquired from a group of animals tested at several time points without exposure (grey shaded area; see Methods; Fig. 3 *F*). Following noise exposure, threshold shifts in UCN3 KO mice were larger when compared to WT mice especially for recovery times of more than 1 day (WT_day0_: 10 (7.8–22.8) dB; UCN3 KO_day0_: 25 (14.5–35.62) dB; *P* = 0.104; WT_day1_: 5 (0–12.0) dB; UCN3 KO_day1_: 15 (10–33.8) dB; *P* ≤ 0.001; WT_day7_: 0 (0–5) dB; UCN3 KO_day7_: 8.5 (5–15) dB; *P* ≤ 0.001; WT_day14_: 0 (–2.1 to 2.5) dB; UCN3 KO_day14_: 7.5 (5–10.6) dB; *P* = 0.017; Kruskal–Wallis one‐way ANOVA on ranks: *P* ≤ 0.001, all pairwise multiple comparison procedures (Dunn's method)). Moreover, in UCN3 KO mice, changes in threshold remained significantly different from baseline even 2 weeks after exposure (Kruskal–Wallis one‐way ANOVA on ranks: *P* ≤ 0.001; multiple comparisons *versus* baseline control group (Dunn's method) at 2 weeks: *P* = 0.014). Conversely, ABR thresholds for WT animals were significantly elevated immediately after exposure but returned to baseline after 1 week of recovery (Kruskal–Wallis one‐way ANOVA on ranks: *P* ≤ 0.001; multiple comparisons *versus* baseline control group (Dunn's method) immediately after exposure: *P* ≤ 0.001 and at 1 week: *P* = 0.325). These data suggest that UCN3 KO mice are more susceptible to long lasting threshold shifts after a single 94 dB SPL noise exposure when compared to their WT counterparts. We next tested whether an even lower intensity, ‘safe’ noise level would elicit noise‐induced hearing loss in UCN3 KO animals.

A single 2 h noise exposure at 84 dB SPL is suggested to result in little or no temporary threshold shifts in normal‐hearing individuals (Gourevitch *et al*. 2014). Indeed, in the WT animals this exposure yielded a threshold shift of only 2.5 dB immediately after exposure, which was not significantly different from baseline (Kruskal–Wallis one‐way ANOVA on ranks; multiple comparisons *versus* baseline control group (Dunn's method): WT_day0_: 2.5 (0–5) dB, *P* = 0.076; WT_day1_: 0 (0–5) dB, *P* = 0.145; WT_day7_: 0 (0–5) dB, *P* = 0.245; WT_day14_: 0 (−3.8 to 0) dB, *P* = 1; Fig. 3 *G*, *I*). The UCN3 KO mice, however, showed significant changes in threshold especially at higher frequencies. Even averaging the dB shift across the whole audiogram showed persistent changes in threshold for at least two weeks after exposure (Kruskal–Wallis one‐way ANOVA on ranks; multiple comparisons *versus* baseline control group (Dunn's method): UCN3 KO_day0_: 10 (7.5–17.5) dB, *P* ≤ 0.001; UCN3 KO_day1_: 6 (2.5–10) dB, *P* ≤ 0.001; UCN3 KO_day7_: 6 (0–10) dB, *P* ≤ 0.001; UCN3 KO_day14_: 5.8 (2.5–10) dB, *P* = 0.003; Fig. 3 *H*, *I*). Additionally, these shifts were greater than those of the WT mice at each time point (Kruskal–Wallis one‐way ANOVA on ranks; multiple comparisons *versus* baseline control group (Dunn's method): day 0: *P* ≤ 0.001; day 1: *P* ≤ 0.001; day 7: *P* = 0.027; day 14: *P* = 0.009; Fig. 3 *I*). These data indicate that noise levels which are considered safe for WT animals cause significant and long‐lasting threshold shifts in mice that lack UCN3 signalling.

### Outer hair cell activity is not impaired in UCN3 KO mice

**Figure 4:**
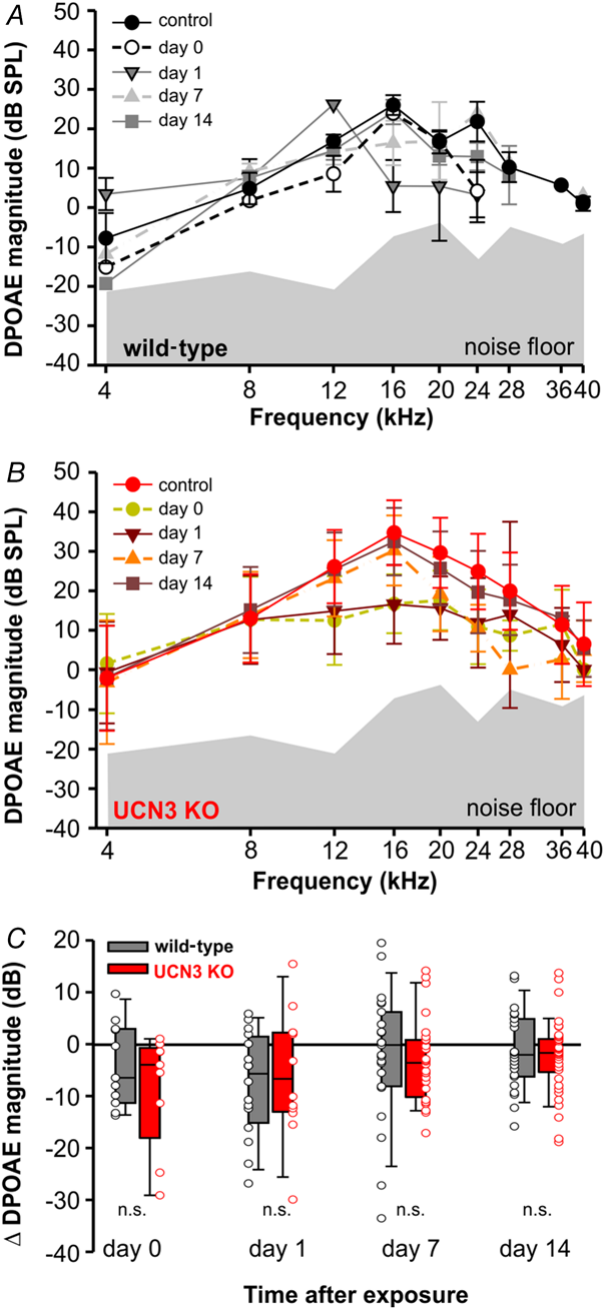
DPOAEs showed a similar amplitude reduction in both genotypes after 94 dB exposure *A* and *B*, grand average and SEM of DPOAE magnitude of cubic distortion products (2*f*_1_ − *f*_2_) at each *f*_2_ at each time before (control), immediately after (day 0), 24 h (day 1) after, 1 week (day 7) and 2 weeks (day 14) after a 2 h/94 dB SPL noise exposure for WT (*A*, black, *n* = 6) and UCN3 KO (*B*, red, *n* = 8). The shaded area shows the noise floor of the recordings. At day 0 and 1 fewer DPOAEs were above the noise floor at the higher frequencies. *C*, changes in DPOAE magnitude relative to pre‐exposure DPOAE level (control) averaged over all frequencies immediately after (day 0), 24 h (day 1) after, 1 week (day 7) and 2 weeks (day 14) after a 2 h/94 dB SPL noise exposure showed no significant differences between genotypes, WT (*A*, black) and UCN3 KO (*B*, red).

Given our results regarding increased susceptibility to and prolonged recovery from noise exposure, we wanted to test whether OHC function was similarly affected. Our histochemical analysis suggested that UCN3 immunoreactivity was most pronounced in areas surrounding IHCs; however, we additionally assessed OHC function using DPOAEs as a metric following our noise exposure protocol. We used the 94 dB SPL exposure and the same time points as in the ABR assessment before. DPOAEs were evoked in WT and UCN3 KO mice across the audiogram (Fig. 4 *A* and *B*). After noise exposure, DPOAE magnitudes were only slightly decreased in both genotypes and recovered along similar timelines (ANOVA: *P* = 0.317; Fig. 4 *A*–*C*). Changes of DPOAE magnitudes of post‐exposure levels compared to pre‐exposure levels (control condition) were calculated and averaged across all frequencies for each animal and plotted in Fig. 4 *C*. Similar to ABRs, frequencies above 8 kHz, which was the lower limit of the noise exposure bandwidth animals were exposed to, were most affected. To conclude, changes in DPOAE magnitude were not significantly different between WT and UCN3 KO animals at any time point (Kruskal–Wallis one‐way ANOVA on ranks: *P* = 0.074). These data therefore suggest that OHC function is not significantly impaired in UCN3 KO animals compared to WT controls.

### Targeted genetic ablation of UCN3‐expressing neurons in the auditory brainstem mimics the UCN3 KO phenotype

**Figure 5:**
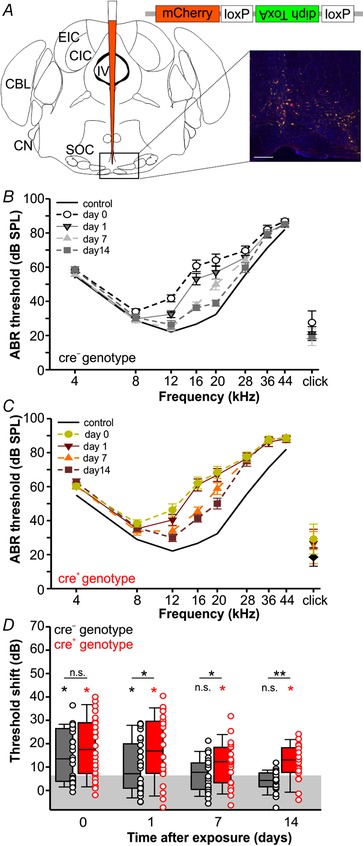
Viral vector mediated targeted ablation of UCN3‐expressing neurons in the brainstem mimics global UCN3 KO phenotype *A*, left, schematic representation of approximate viral injection site in the brainstem. Right, confocal image of boxed area in schematic representation showing neurons (cre^−^, UCN3^−^) transfected with the virus (red) which survived due to lack of diphtheria toxin production (scale bar = 200µm). MAP2 counterstain in blue. *B* and *C*, audiograms of virus‐injected cre^−^ littermates (*n* = 26) (*B*) and cre^+^ littermates (*n* = 26) (*C*) at different time points following noise exposure of 2 h/94 dB SPL compared against age‐matched naïve (no noise exposure) controls. *D*, threshold shift in dB relative to controls (black continuous line) averaged over all frequencies following noise exposure in cre^−^ (black) and cre^+^ (red) mice. Lower black and red asterisks indicate that a significant difference was detected relative to baseline (shaded area). Upper asterisks indicate whether a significant difference was found between genotypes: Kruskal–Wallis one‐way ANOVA on ranks followed by multiple comparisons *versus* Control group (Dunn's method).

Besides UCN3 expression in the auditory brainstem, UCN3 positive neurons are present in other brain regions involved in regulation of the body's stress axis (Deussing *et al*. 2010). To test whether the effects of UCN3 in recovery from acoustic trauma are independent of other, more typically stress‐related brain areas, targeted genetic ablation of UCN3‐expressing neurons in the auditory brainstem was performed. Stereotaxic injections of recombinant adeno‐associated virus (AAV) expressing the cre‐dependent diphtheria toxin A fragment (rAAV8/EF1α‐mCherry‐Flex‐dtA: AAV‐DTA) into the superior olivary complex (SOC) of the UCN3‐tdTomato mouse line (Fig. 5 *A*) enabled targeted ablation of UCN3 positive brainstem neurons without affecting UCN3‐expressing neurons in other brain areas. Viral injections were performed on entire litters born to UCN3‐cre × Ai9‐flox (tdTomato) breeders. The offspring of these breeding pairs included pups of which some expressed cre‐recombinase in UCN3 neurons (cre^+^) and others did not (cre^−^). Histological examination was used to confirm genotypes and was not carried out until after all noise exposure and ABR recordings were completed and analysed so that the experimenter was blind to the effect of the virus injection. A month following viral infection, the ABR and noise exposure (94 dB SPL) protocols were performed and hearing thresholds were assessed at day 0, 1, 7 and 14 following exposure. The resulting audiograms are plotted in Fig. 5 *B* and *C* for cre^−^ and cre^+^ mice, respectively. Shifts in auditory thresholds following noise exposure are shown in Fig. 5 *D* for the cre^−^ animals and the cre^+^ animals. Both sets of animals experienced similar threshold shifts immediately following noise exposure (cre^−^_day0_: 13.5 (3.9–26.4) dB; cre^+^_day0_: 17.5 (7.2–28.9) dB; *t* test: *P* = 1.000; Fig. 5 *D*), but auditory thresholds in the cre^−^ animals recovered faster than in the cre^+^ animals and were no longer significantly different from the baseline at the 7 and 14 day time points (Kruskal–Wallis one‐way ANOVA on ranks: *P* ≤ 0.001; multiple comparisons *versus* baseline control group (Dunn's method): cre^−^_day0_: 13.5 (3.9–26.4) dB, *P* ≤ 0.001; cre^−^_day1_: 7.1 (0.9–19.9) dB, *P* = 0.004; cre^−^_day7_: 5.6 (−0.4 to 11.6) dB, *P* = 0.116; cre^−^_day14_: 4.2 (1.5–7.5) dB, *P* = 0.523; Fig. 5 *E*). In contrast, auditory thresholds in cre^+^ animals recovered only slowly and remained different from baseline even after 2 weeks (Kruskal–Wallis one‐way ANOVA on ranks: *P* ≤ 0.001; multiple comparisons *versus* baseline control group (Dunn's method): cre^+^_day0_: 17.5 (7.2–28.9) dB, *P* ≤ 0.001; cre^+^_day1_: 16.9 (7.2–29.6) dB, *P* ≤ 0.001; cre^+^_day7_: 12.3 (3.3–18.4) dB, *P* ≤ 0.001; cre^+^_day14_: 12.3 (4.9–18.0) dB, *P* ≤ 0.001). This maladaptive recovery from mild acoustic trauma in cre^+^ mice was reminiscent of the UCN3 KO phenotype (Fig. 3 *F* and *I*) and suggests a protective role for local UCN3‐expressing neurons in the auditory brainstem.

## Discussion

Previous studies suggest that alterations in corticotropin releasing factor signalling results in abnormal auditory function (Graham *et al*. 2010; Graham & Vetter, 2011; Graham *et al*. 2011; Basappa *et al*. 2012). In the present study, we demonstrate that intact UCN3 signalling in the auditory pathway contributes to the prevention of age‐ and noise‐induced hearing loss. We show that global as well as brainstem‐specific genetic reduction of UCN3 signalling caused maladaptive stress responses following moderate noise exposure.

### UCN3 expression in the cochlea and brainstem

UCN3 is a neuromodulatory peptide that is immediately shuttled to neuronal terminal regions following translation, resulting in low concentrations in the cell body (Bittencourt *et al*. 1999). This and the small size of the peptide require special protocols for UCN3 antibody staining including ventricular colchicine injection in order to trap and identify the peptide in neuronal somata (Li *et al*. 2002). We therefore used a different visualization approach and employed a UCN3‐tdTomato reporter line (Shemesh *et al*. 2016) to determine the location of UCN3‐expressing neurons and terminals in the lower auditory system (Li *et al*. 2002). Additional immunohistochemical markers were used to characterize the cell types and location of UCN3‐expressing cells and terminals in more detail.

UCN3 positive neurons were commonly observed in the auditory nerve root region and towards the posteroventral cochlear nucleus. A subset of the VCN UCN3 positive neurons were globular bushy cells as indicated by the presence of tdTomato‐labelled calyx terminals in the lateral MNTB (Spirou *et al*. 1990). Another population of UCN3 positive VCN neurons show strong projections into the dorsal cochlear nucleus and could therefore be multipolar stellate cells (Doucet & Ryugo, 1997). Interestingly, multipolar cells of the posteroventral cochlear nucleus have also been shown to provide afferent input to efferent neurons in the SOC (Thompson & Thompson, 1991; Brown *et al*. 2013), which could be one way in which UCN3 signalling can unfold its protective function. In this study, we observed labelled axons and terminals in the VNTB and the LSO cell area, suggesting that neurons in the MOC and LOC efferent systems may be regulated via presynaptic UCN3 signalling. However, the results of our DPOAE recordings suggest that the origin of the genotype‐specific difference in recovery from mild acoustic trauma may reside in the LOC‐IHC system, since OHC activity was not different between both genotypes (Fig. 4 of this study).

In the cochlea itself, UCN3 expression was evident as puncta near the base of the IHCs implying that UCN3 is expressed either in the postsynaptic afferent fibre or in the presynaptic terminal of the LOC efferents, which form synapses onto the primary afferents. Retrograde labelling from the cochlea to the auditory brainstem revealed many neuronal somata within the periolivary nuclei of the SOC including the SPN and the DPO (Thompson & Thompson, 1991) in guinea pig. Other studies have suggested that the SPN was an area of dense UCN3 immunoreactivity (Li *et al*. 2002); however, we observed a limited number of UCN3^+^ cells in the SPN in our UCN3‐tdTomato reporter mouse and instead saw a population of UCN3^+^ cells in an area between the MNTB and SPN and also in the DPO. While many neurons in the periolivary regions like SPN (in guinea pigs) and DPO (in other rodents) can be retrogradely labelled via cochlear injections (Thompson & Thompson, 1991; Warr *et al*. 1997), these neurons do not always exhibit the strong cholinergic labelling that typically identifies efferents of the LOC and MOC (Vetter & Mugnaini, 1992; Darrow *et al*. 2006; Brown & Levine, 2008). A group of these atypical LOC neurons called ‘shell’ neurons project back to the cochlea to innervate mainly the modiolar side of IHCs (Vetter & Mugnaini, 1992; Warr *et al*. 1997), a similar staining pattern as seen in the UCN3‐tdTomato mouse line of the present study. Based on our results we propose that a subpopulation of efferent neurons in the SOC and DPO may use UCN3 as their neuromodulatory peptide instead and provide the ligand to the cochlear CRF‐R2 (Graham *et al*. 2010) via a local pathway that is well suited to integrate activity‐dependent changes in the auditory system without involving the whole HPA axis. Such a localized, activity‐dependent component to cochlear protection has been described by a study performing acoustic conditioning before potentially harmful noise exposure (Yamasoba *et al*. 1999).

### Cellular mechanisms of UCN3 signalling

UCN3 is a high‐affinity ligand of CRF‐R2. While CRF‐R1 is associated with the initiation of the body's responses to stress (Gallagher *et al*. 2008), CRF‐R2 activation is suggested to promote the recovery from stressful situations (Neufeld‐Cohen *et al*. 2010). Both receptor types are metabotropic receptors, so that their action will strongly depend on whether the receptor is present pre‐ or postsynaptically as well as on the targets of the second messenger cascades initiated by the G‐protein. For example, activation of CRF‐R1 on glutamatergic presynaptic terminals potentiates excitatory postsynaptic currents, while in the same neuron activation of CRF‐R2 on GABAergic presynaptic terminals attenuates the net excitatory post‐synaptic currents (Williams *et al*. 2014). In addition to UCN3 action on transmitter release, the effect of UCN3 on postsynaptic CRF‐R2 appears to be specific to the type of neuron it is expressed in and a variety of different effects have been described accordingly. In neurons of the posterior bed nucleus of the stria terminalis and neurons of the medial amygdala, the application of UCN3 resulted in more depolarized resting membrane potentials, an increase in input resistance and an increase in the neurons’ firing rates (Henckens *et al*. 2016; Shemesh *et al*. 2016). In a different study, bath application of UCN3 hyperpolarized neurons in the paraventricular nucleus of the hypothalamus based on the subsequent activation of an inwardly rectifying potassium channel (Chu *et al*. 2013). This hyperpolarization enhanced excitability in parvocellular neurons, but decreased excitability in magnocellular neurons (Chu *et al*. 2013). As a neuropeptide of a very small size, UCN3 is packed into dense core vesicles (van den Pol, 2012) and can then be released by either kiss‐and‐run or full fusion depending on the form of stimulation (Zhang *et al*. 2011). The sensor of activity levels which decides the mode of vesicle fusion is suggested to be synaptotagmin (Zhang *et al*. 2011), which is abundantly expressed in the cochlea (Johnson *et al*. 2013), in auditory brainstem neurons (Moritz *et al*. 2015) and also in the calyx of Held (Young & Neher, 2009; Luo & Sudhof, 2017). In future studies we will target the physiology of this receptor system in the auditory pathway to reveal the cellular mechanisms underlying the effects of UCN3 on stress recovery described in the present study.

### UCN3 signalling contributes to stress recovery and restoration of homeostasis

The temporary threshold shift that occurs following acoustic trauma will usually decline after a period of about 24 h (Fuente, 2015). Such recovery from stress malfunctions in mice that lack the urocortins as endogenous ligands to the CRF‐R2 (Neufeld‐Cohen *et al*. 2010). In the present study we show that while WT mice suffered only from a temporary threshold shift following 94 dB SPL noise exposure, in UCN3 KO mice the threshold shift was persistent at least up to 2 weeks post‐exposure. A similar phenotype was observed in mice following targeted ablation of UCN3 positive cells in the auditory brainstem. While the magnitude of the threshold shifts was not large at the end of the recovery period, one can imagine that these minor shifts could accumulate over a lifetime of repeated acoustic insult and lead to significant hearing loss. Even without insult *per se*, we observed this in our ageing study where UCN3 KO mice showed a more rapid rate of hearing loss. These results suggest that elimination of UCN3 either globally (KO) or specifically in the brainstem (cre^+^ mice in this study) can lead to deficits in recovery from acoustic noise‐induced stress, similar to the function of UCN3 in the HPA axis (Coste *et al*. 2000; Neufeld‐Cohen *et al*. 2010, 2012; Henckens *et al*. 2016; Shemesh *et al*. 2016).

### CRF‐R activity and auditory sensitivity

Normal activity of the CRF‐R system has been shown to influence thresholds and development of the auditory system. Genetic manipulations of the two receptor types in this system have differing effects. Knockout of CRF‐R1 causes increased auditory thresholds and abnormal development of synaptic morphology in hair cells of naïve mice (Graham *et al*. 2011). The importance of CRF‐R1 for normal auditory development is corroborated by a strong transient expression of the CRF‐R1 ligand UCN1 just around hearing onset (Kaiser *et al*. 2011). In contrast, CRF‐R2 knockout mice showed lower thresholds when raised in low noise conditions (Graham *et al*. 2010). In our study, audiograms of naïve UCN3 KO mice were not significantly different from wild‐type animals at most frequencies, but tended to have lower thresholds, similar to the CRF‐R2 knockouts (Graham *et al*. 2010). The similarity of the audiograms of naïve WT and UCN3 KO animals may indicate the presence of compensatory mechanisms acting to mitigate the lack of UCN3 signalling during development. Our viral injection experiments were designed to (a) circumvent this compensation and (b) distinguish between the actions of UCN3 positive neurons in other parts of the brain outside the auditory brainstem. The fact that we still observed a maladaptive recovery from the 2 h noise exposure to 94 dB SPL points towards a protective role of UCN3‐expressing neurons in the auditory brainstem of mature mice. The UCN3 positive brainstem neurons may either be part of efferent neuronal populations which project back to the cochlea and modulate cochlear sensitivity or they could modulate the efferent neurons via a presynaptic action of UCN3. Future work will focus on detailed characterization of UCN3 synaptic physiology.

## Additional information

### Authors’ present addresses

M. J. Fischl: National Institutes of Health, Section on Neuronal Circuitry, 35A Convent Dr., Bethesda, MD 20814, USA.

J. L. Sinclair: Ear Institute, University College London, London, UK.

### Competing interests

The authors declare no competing financial interests.

### Author contributions

The experiments were carried out at the Biocenter of the Ludwig‐Maximilians‐University. All authors contributed to conception or design of the work, acquisition, analysis or interpretation of data, and drafting the work or revising it critically for important intellectual content. All authors confirm that they approved the final version of the manuscript, agree to be accountable for all aspects of the work in ensuring that questions related to the accuracy or integrity of any part of the work are appropriately investigated and resolved, and all persons designated as authors qualify for authorship, and all those who qualify for authorship are listed.

### Funding

This research was supported by the German Research Council (KO2207/3‐1, SFB870/2‐A10 and GS‐82 Graduate School of Systemic Neurosciences GSN^LMU^), German Ministry of Science and Education (Grant No 01EO1401).
